# Case report: Clinical course and treatment of SARS-CoV-2 in a pediatric CAR-T cell recipient with persistent hypogammaglobulinemia

**DOI:** 10.3389/fped.2023.1076686

**Published:** 2023-03-10

**Authors:** Howard Sanders, Christina Callas, Helaine St. Amant, Jong Chung, Victoria R. Dimitriades, Natasha A. Nakra

**Affiliations:** ^1^ Department of Pediatrics, University of California Davis Medical School, Sacramento, CA, United States; ^2^ Division of Pediatric Hematology & Oncology, Department of Pediatrics, University of California Davis Medical School, Sacramento, CA, United States; ^3^ Division of Pediatric Allergy & Immunology, Department of Pediatrics, University of California Davis Medical School, Sacramento, CA, United States; ^4^ Division of Pediatric Infectious Diseases, Department of Pediatrics, University of California Davis Medical School, Sacramento, CA, United States

**Keywords:** COVID-19, SARS-CoV-2, hypogammaglobulinemia, CAR-T cell therapy, virus neutralizing monoclonal antibody, remdesivir, pediatric

## Abstract

This report describes a pediatric patient who underwent chimeric antigen receptor (CAR) T-cell therapy for refractory B-cell acute lymphoblastic leukemia (B-ALL) four years prior, with resultant hypogammaglobulinemia for which he was receiving weekly subcutaneous immune globulin. He presented with persistent fever, dry cough, and a tingling sensation in his toes following a confirmed COVID-19 infection 3 weeks prior. His initial nasopharyngeal SARS-CoV-2 PCR was negative, leading to an extensive workup for other infections. He was ultimately diagnosed with persistent lower respiratory tract COVID-19 infection based on positive SARS-CoV-2 PCR from bronchoalveolar lavage (BAL) sampling. He was treated with a combination of remdesivir (antiviral) and casirivimab/imdevimab (combination monoclonal antibodies) with immediate improvement in fever, respiratory symptoms, and neurologic symptoms.

## 1. Introduction

Intact humoral immunity is critical to successful elimination of SARS-CoV-2 infection, as well as for prevention of re-infection (1). Prior studies have indicated that SARS-CoV-2 infected individuals who have recovered from infection produce immunoglobulin (Ig)G antibodies targeting the viral N (nucleocapsid) and S (spike) proteins, including the receptor-binding domain. Immunocompromised individuals with iatrogenic B cell depletion and humoral immunodeficiencies associated with dysregulation are at higher risk of severe COVID-19 infection (2).

Secondary hypogammaglobulinemia is a known sequela of chimeric antigen receptor (CAR) T-cell therapy, an immunotherapy used in the treatment of hematologic malignancies, including relapsed or refractory pediatric B-cell acute lymphoblastic leukemia (B-ALL). CAR-T therapy targets malignant B-cells by engineering anti-CD19 CAR T cells that are intended to destroy malignant cells but also commonly destroy normal B-cells (3). The resulting deficiency can persist for several years after therapy (4). Due to the increased risk for life-threatening infections, it is recommended that pediatric patients with hypogammaglobulinemia following CAR T-cell therapy receive routine intravenous or subcutaneous immunoglobulin G (5).

This case report describes the clinical course of persistent SARS-CoV-2 infection in a 13-year-old child with a history of B-ALL with secondary hypogammaglobulinemia following CAR-T therapy.

## 2. Case description

A 13-year-old male child with history of B-ALL who had been in remission for four years following CAR-T cell therapy presented with two weeks of malaise and fevers following COVID-19 infection 23 days earlier. At the time of initial COVID-19 diagnosis, he had nasal congestion, mild cough, and dysgeusia without fever. Multiple family members also tested positive for COVID-19 at that time. Diagnosis was made *via* nasopharyngeal (NP) swab which was positive for SARS-CoV-2 by polymerase chain reaction (PCR).

### 2.1. Prior malignancy, hypogammaglobulinemia treatment, and infection history

At time of his B-ALL diagnosis, initial cytogenetics were concerning for hypodiploidy (associated with a poor prognosis). He had refractory disease and was referred for hematopoietic stem cell transplant (HSCT), which he received 6 months after his initial diagnosis with a matched sibling donor. Unfortunately, he suffered relapse shortly after HSCT. He ultimately underwent CAR-T therapy 9 months later after which he entered remission. Bridging therapies he received prior to CAR-T included blinatumomab, which is a bispecific CD19-directed CD3 T-cell engaging immunotherapy. Following CAR-T therapy, he developed B cell aplasia and secondary hypogammaglobulinemia requiring immunoglobulin replacement.

Baseline immunological evaluation three years prior to the current admission noted a normal complete blood count with absolute lymphocyte count of 3800 cells/ul. IgG, IgA, and IgM were all two standard deviations below normal with a history of intermittent use of IgG supplementation due to low levels. Lymphocyte enumeration revealed elevated numbers of T cells (mostly due to increased CD8 cells), along with absent B cells. Mitogen proliferation was normal and T-cell receptor (TCR) spectratyping showed a normal distribution of the T cell repertoire. He was initiated on monthly IVIg (intravenous immune globulin) supplementation (20 grams) and eventually transitioned to weekly Cuvitru (5 grams) subcutaneous injections without an issue. Repeat lymphocyte subset testing 3 months prior to admission continued to demonstrate absence of B cells (Table 1).

**Table 1:** Lymphocyte subset testing prior to presentation.

Labs	Normal value	Patient's value
Lymphocyte subsets
CD3+ %	52%–90%	90
CD3 + absolute count	850–3200/µl	2763
CD3 + CD4+ %	20–65%	45
CD3 + CD4 + absolute count	400–2100/µl	1378
CD3 + CD8+ %	14%–40%	43
CD3 + CD8 + absolute count	300–1300/µl	1337
CD4/CD8 ratio	0.9–3.4	1.0
CD19+ %	7%–24%	<1
CD19 + absolute count	120–740/µl	<8
CD3-CD56+ %	4%–51%	10
CD3-CD56 + absolute count	92–1200/µl	295

He had normal pulmonary function testing (PFT) two years after his initial B-ALL diagnosis, and again three months prior to admission.

Since his B-ALL diagnosis, he had a documented history of three viral infections: rhinovirus/enterovirus on two separate occasions, and influenza A once for which he was treated with a five day course of oseltamivir as an outpatient. He did not have a history of any significant bacterial or fungal infections.

### 2.2. Hospital course

Three weeks after initial COVID diagnosis, the patient presented to the emergency department with fevers for two weeks, which were initially intermittent, but had become constant for five days prior to admission to the hospital. He reported worsening cough, although he denied shortness of breath, wheezing, or chest pain. He noted ten pounds of unintentional weight loss since symptom onset. While in the emergency room, he started complaining of tingling and pain in his toes. There was no history of recent travel, animal exposures or other infectious exposures.

On initial physical exam, the patient had a temperature of 37.4°C, a pulse of 130 beats/minute, blood pressure 116/71 mm Hg, respiratory rate 26 breaths/minute, and oxygen saturation 98% on room air. Two hours later, he developed a fever to 38.2°C. He did not appear to be in distress and was alert and oriented. His work of breathing was normal and breath sounds were mildly diminished in the left lower and right middle lung fields. Capillary refill was less than two seconds, and there were no rashes, ecchymoses, or petechiae. Neurological assessment revealed normal cranial nerve exam, normal strength in all 4 extremities, normal deep tendon reflexes, and intact sensation. However, he was exquisitely tender to touch on both the dorsal and plantar aspects of his toes on the left foot. With reported tingling in his bilateral toes and hyperalgesia with palpation of the bilateral distal lower extremities in a stocking-glove distribution, a clinical diagnosis of peripheral neuropathy was made.

Initial laboratory testing revealed a normal complete blood count with elevated inflammatory markers (Table 2). Notably, point-of-care PCR testing for influenza A and B and SARS-CoV-2 from a NP swab were negative. A chest x-ray demonstrated bilateral patchy peripheral opacities, left greater than right. The patient was admitted to the pediatrics inpatient service for additional workup and management and was started on IV ceftriaxone for treatment of presumed community-acquired pneumonia following recent SARS-CoV-2 infection. A blood culture drawn on admission resulted as negative after 5 days incubation.

**Table 2:** Laboratory findings at the time of presentation.

Labs	Normal value	Patient's value
White blood cell count, × 10^9^/L	4.5–13.5	8.9
Neutrophil count, × 10^9^/L	1.8–8.0	5.2
Lymphocyte count, × 10^9^/L	1.5–6.5	3.3
Hemoglobin (g/dl)	11.5–15.5	11.7
Platelet count, × 10^9^/L	130–400	193
ALT (U/L)	6–63	35
Albumin (g/dl)	3.8–5.4	2.9
Erythrocyte sedimentation rate (mm/hr)	<15	67
C-reactive protein (mg/dl)	<0.8	7.8
Lactate dehydrogenase (U/L)	90–200	418
Ferritin (ng/ml)	22–322	5,665
Troponin T (ng/L)	<6	<6
Brain natriuretic peptide (pg/ml)	<25	<25
aPTT (sec)	29–40	31.4
INR	0.87–1.18	1.20
D-dimer (ng/ml)	0–230	432
Fibrinogen (mg/dl)	154–448	609
IgG (mg/dl)	528–2190	671
IgA (mg/dl)	44–395	8
IgM (mg/dl	56–352	<25

Abbreviations: dl, deciliter; g, gram; hr, hour; Ig, immunoglobulin; L, liter; ml milliliter; mm, millimeter; ng, nanogram; pg, picogram; sec, second; U, unit.

**Figure 1:**
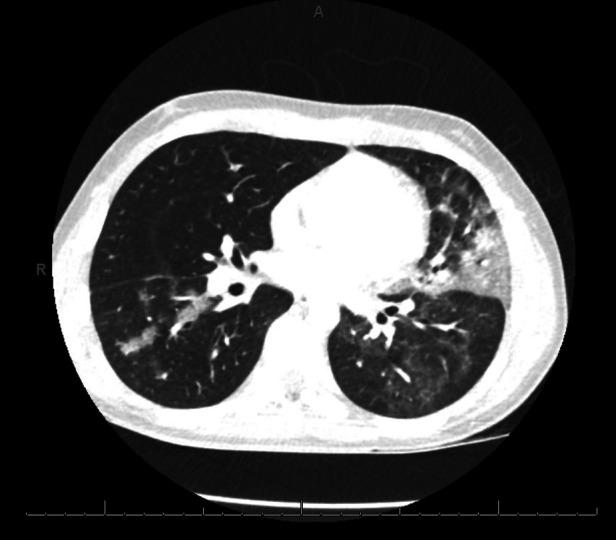
The patient underwent a computed tomography (CT) scan of the chest on hospital day (HD) 4 which revealed “bilateral patchy consolidations with adjacent, scattered ground glass opacities”.

Following admission, he exhibited daily fevers ranging from 38.4–40.2°C as well as a persistent dry cough. Due to concern for multisystem inflammatory syndrome in children (MIS-C) following COVID-19 infection, a SARS-CoV-2 serology was obtained and was negative, with the caveat that antibody testing was not considered reliable given his known hypogammaglobulinemia. Daily labs were obtained which demonstrated persistently elevated C-reactive protein (CRP) and rising ferritin. A computed tomography (CT) scan of the chest was performed on hospital day (HD) 4 which revealed “bilateral patchy consolidations with adjacent, scattered ground glass opacities (Figure 1).” Antimicrobial coverage was broadened from ceftriaxone (50 mg/kg every 24 h) to vancomycin (15 mg/kg every 8 h, increased to every 6 h following the fourth dose based on trough level), cefepime (50 mg/kg every 8 h), and micafungin (150 mg every 24 h). Evaluation for other infectious etiologies was unrevealing, including common bacterial pathogens (*Streptococcus pneumoniae*, *Legionella pneumophila*), viral pathogens (EBV, CMV, parvovirus) and fungal pathogens (Cryptococcus, Aspergillus). Adenovirus blood PCR was positive at a low level that was not thought to be elevated enough to explain his symptomatology and rather reflected reactivation. Indirect testing for fungal pathogens returned with very elevated beta-D-glucan (>500) which was attributed to IVIg administration (known to falsely elevate this value). Subsequent labwork revealed decreasing CRP, however fevers persisted and ferritin levels continued to increase. Echocardiogram done on HD 4 showed low-normal left ventricular function and normal coronary arteries. He was changed to meropenem (700 mg every 8 h) and azithromycin (10 mg/kg every 24 h) on HD 5 after a sputum gram stain revealed gram negative bacilli. However, sputum culture later grew normal upper respiratory flora without a predominant organism. As he was due for his home dosing of weekly subcutaneous Ig, he was given a monthly dose of IVIg (20 g) on HD 5.

Due to persistent symptoms, bronchoscopy was performed on HD 11, during which turbid bronchioalveolar lavage (BAL) fluid was obtained for further testing. Cell count on BAL fluid revealed 1,850 WBC with a differential of 70% lymphocytes, 18% histiocytes, and 6%neutrophils. Infectious evaluation from samples obtained during the bronchoscopy was unrevealing—negative bacterial and fungal cultures, ova and parasites (O & *P*) examination, acid-fast bacilli (AFB) smear and culture, *Pneumocystis jirovecii* direct-fluorescent antibody, respiratory pathogen panel, and *Aspergillus* galactomannan. Flow cytometry was performed on BAL fluid and was not consistent with relapsed malignancy. SARS-CoV-2 PCR and viral cultures were not performed given the negative SARS-CoV-2 NP PCR done on admission.

Due to the patient's oncologic history, persistent fevers, and increasing ferritin, etiologies other than infection were considered, including relapsed leukemia and associated hemophagocytic lymphohistiocytosis (HLH). Lymphocyte enumeration was performed again and did not demonstrate the presence of B cells or blast forms. Bone marrow biopsy was performed on HD 18 and did not reveal a monotypic B cell population, increased blasts, or hemophagocytosis. Repeat TCR spectratyping did not reveal any changes in the normal distribution of T cell receptor families.

**Figure 2:**
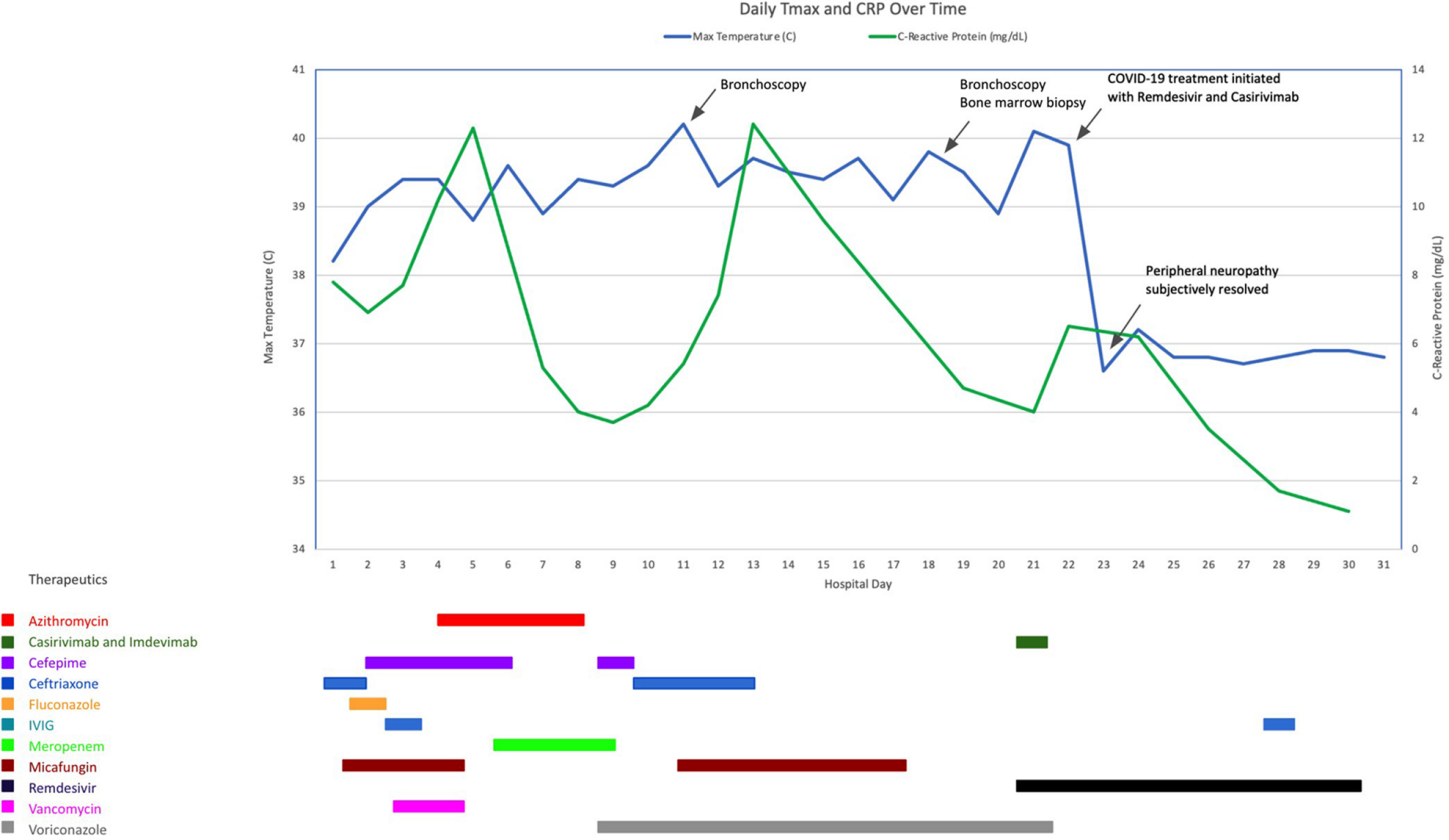
Clinical course and treatment of the patient including daily maximum temperature (Tmax) and C-reactive protein (CRP) during his hospitalization.

Chest x-ray on HD 17 demonstrated a new right upper lobe infiltrate; thus a second bronchoscopy was also performed on HD 18, with additional infectious studies sent and transbronchial biopsy performed. SARS-CoV-2 PCR performed on BAL fluid from this bronchoscopy was positive after 20 cycles of PCR, indicative of a high viral load (6). Of note, a simultaneous SARS-CoV-2 PCR on an NP swab specimen resulted as negative. After multi-disciplinary discussion, the patient was treated with an infusion of combination virus-neutralizing monoclonal antibodies (mAb) against SARS-CoV-2 (casirivimab/imdevimab 1,200 mg/1,200 mg) that were active against the circulating strain of SARS-CoV-2 (delta variant), as well as a 10-day course of remdesivir (200 mg loading dose followed by 100 mg daily). The patient defervesced within hours of initiation of treatment with casirivimab/imdevimab and remdesivir. Dexamethasone was avoided due to concern that this could worsen infection by suppressing T cell activity. He improved subjectively, with self-reported increase in energy and resolution of his bilateral foot pain within 24 h. Figure 2 demonstrates his fever curve and CRP levels in relation to treatment.

## 3. Discussion

Since the emergence of SARS-CoV-2 in 2019, most published studies suggest that the majority of healthy children who contract COVID-19 are asymptomatic or have a mild-to-moderate disease course compared to adults (7). Data regarding outcomes of immunocompromised children infected with SARS-CoV-2 are more limited, and experts suggest that treatment should be decided on a case-by-case basis depending on the underlying immune defect (8). Here we describe a unique case of prolonged COVID-19 lower respiratory tract infection in a pediatric patient who was immunocompromised due to prior CAR-T therapy for B-ALL with resultant B cell aplasia and hypogammaglobulinemia. Our patient also exhibited peripheral neuropathy as an acute clinical manifestation of COVID-19 infection which is a relatively unusual finding. Finally, our patient's clinical course was complicated by a negative SARS-CoV-2 nasopharyngeal PCR swab, which raised questions of alternative diagnoses (e.g., relapsed leukemia, hemophagocytic lymphohistiocytosis) and ultimately delayed the initiation of appropriate antiviral therapy. He was ultimately diagnosed with SARS-CoV-2 by PCR tests run on BAL fluid. Generally there is good concordance between NP and BAL samples, although discordant results have been described (9). It is also interesting to note that at his initial infection three weeks prior to admission, our patient tested positive by PCR testing *via* NP swab, indicating that it was previously possible to identify infection by this modality for him.

Chimeric antigen receptor (CAR) T-cell therapy is an immune-modulating treatment approved in the pediatric population for refractory and relapsed B-ALL. CAR T-cells are autologous T cells that are removed from a patient, engineered with anti-CD19 + chimeric antigen receptors that combine an antigen recognition domain with T-cell activation domains, and then re-infused to the patient. The intended result is destruction of malignant CD19+ B cells, although B cell aplasia and subsequent hypogammaglobulinemia secondary to destruction of normal B cells is a common side effect (“on-target, off-tumor”). Response to CAR-T cell therapy in pediatric and young adult patients with refractory and relapsed B-ALL is generally favorable and durable, with 63% overall survival rate at 36 months, and an estimated 9.28 quality-adjusted life-years (QALYs) gained (10, 11). Survival in relapsed pediatric B-ALL patients with measurable residual disease (MRD) positivity following re-induction who are treated with CAR-T cell therapy prior to HSCT has been shown to improve survival compared to patients treated with chemotherapy prior to HSCT (12).

Patients who undergo CAR-T cell therapy are at increased risk of infection due to multiple factors including prior chemotherapy and cancer treatment, lymphodepleting chemotherapy during the CAR-T cell process, cytokine release syndrome in the immediate post CAR-T cell infusion time period, and hypogammaglobulinemia (13). In children, infections with bacteria predominate in the first 28 days after CAR-T therapy, following which respiratory viral infections are more common. The majority of these respiratory viral infections are considered mild-moderate and not life-threatening (14).

Hypogammaglobulinemia is a common complication after CAR-T cell therapy, with one study demonstrating that 29% of patients had low IgG levels at 63 days post-CAR T-cell infusion (14). Of note, hypogammaglobulinemia is more common and more severe in pediatric as compared to adult patients and can persist for >4 years post infusion (5, 15). Some experts suggest that pediatric CAR T cell recipients with IgG levels less than 400 mg/dl should receive regular supplementation of immune globulin.

Persistent SARS-CoV-2 infection in patients with B cell deficiency, including CAR-T cell recipients, has been described. In one report, 2 of 3 CAR-T cell recipients had prolonged infection lasting >5 months (16). In another case report, a 73-year-old patient with multiple myeloma presented 25 days after CAR-T therapy with cough and hypoxia, and was found to be positive for SARS-CoV-2 by RT-PCR testing of an NP swab with a low cycle threshold (20.1 for nucleocapsid protein, 21.5 for envelope protein) (17). He was treated with convalescent plasma and remdesivir and improved, but subsequently presented again at 41 days post CAR-T therapy with cough and dyspnea, progressing to hypotension and intubation on day 55. At that time, he was still positive for SARS-CoV-2 from NP swab with a low cycle threshold (13.3 for N1 gene; 16 for E gene). He was treated again with convalescent plasma as well as dexamethasone but died on day 74 due to respiratory failure. SARS-CoV-2 RNA was retrospectively detected in his blood plasma samples with an increase observed after the steroid course. Failure of convalescent plasma treatment was attributed to multiple factors including (1) concomitant T cell deficiency limiting immune response (2) possible low levels of effective anti- SARS-CoV-2 antibodies in the convalescent plasma (3) intra-host evolution of SARS-CoV-2 allowing for evasion from circulating antibodies and (4) steroid treatment which may have contributed to T cell dysfunction. In fact, SARS-CoV-2 intra-host evolution in patients with chronic infection has been well described and may account for emergence of new variants (18).

Larger studies of adults who have undergone CAR-T cell therapy report high mortality and morbidity secondary to COVID-19 infection. A multicenter study from Europe of 56 patients who had undergone CAR-T therapy at a median of 7.4 months prior to COVID-19 diagnosis (range 1 day to 25.3 months), reported a 41.1% attributable mortality rate. Additionally, 80% of patients required admission to the hospital for COVID-19 infection with 39.3% requiring admission to the ICU. Of note, there was only a single pediatric patient in that study (19). Factors associated with mortality included older age, not being in complete remission at the time of COVID-19 diagnosis, and having metabolic comorbidities, such as diabetes and cardiovascular disease. The median time to clinical resolution of COVID-19 infection was reported to be 20 days (range 0–157 days). Patients were treated with varying combinations of convalescent plasma, steroids, and remdesivir, although no significant impact of these therapies on clinical outcome was found.

Once the diagnosis of SARS-CoV-2 was made, our patient was treated with both casirivimab/imdevimab (virus-neutralizing monoclonal antibody) and remdesivir (antiviral), and symptoms completely resolved within approximately 24 h of initiation of treatment. Virus-neutralizing mAb products have typically been used for patients with mild-moderate SARS-CoV-2 infection who are at risk for progression to severe disease (20). However, use of mAb in combination with remdesivir for patients with severe B cell deficiency and protracted SARS-CoV-2 infection has been described previously in 3 adult patients. Combination mAb therapy is preferred over a single monoclonal antibody to prevent emergence of mutant virus, and remdesivir is used adjunctively to decrease viral burden (21). This therapeutic approach was also utilized in an adult patient with X-linked agammaglobulinemia on chronic immunoglobulin replacement who was reported to have a recurrent disease course, with initial admission for COVID-19 pneumonia with hypoxia and treatment with remdesivir, dexamethasone, IVIG, and antibiotics, followed by re-admission two weeks later for fever and diarrhea with negative SARS-CoV-2 RT-PCR testing by NP swab. He was ultimately diagnosed with persistent SARS-CoV-2 infection on day 30 of hospitalization (positive RT-PCR testing from sputum and NP swab) and treated with a 10-day course of remdesivir (days 31–40) with immediate improvement. He was also given monoclonal antibodies (casirivimab/imdevimab) on day 38 of hospitalization and tolerated the infusion well (22).

Our patient's clinical course was complicated by clinical symptoms consistent with a peripheral neuropathy which was initially quite perplexing. Given the complete resolution within one day of initiation of anti-SARS-CoV-2 therapies, we concluded that this symptom was secondary to COVID-19 infection as well. While there is limited literature describing Acute Neuropathy Associated with Covid-19 (or ANAC-19), it appears that the majority of affected patients develop neurologic symptoms within one month of infection (23). Common clinical symptoms include paraparesis, quadriparesis, cranial nerve involvement, and hyporeflexia, although sensory symptoms have also been reported.

Overall, our case demonstrates a prolonged and atypical course of COVID-19 in an immunocompromised pediatric patient with B cell aplasia and hypogammaglobulinemia following CAR-T cell therapy. It additionally highlights unique and uncommon features of COVID-19 infection (e.g., acute neuropathy) and the consideration of a variety of treatment options in an immunocompromised host. The use of combination monoclonal antibody treatment with an antiviral medication was highly efficacious for our patient and has been used in similar clinical contexts in adult patients. We avoided the use of corticosteroid treatment due to concern that T cell function may be affected, as prior studies have indicated that the presence of both B and T cell dysfunction may portend a poorer prognosis as compared to patients with a pure B cell deficiency.

## Data Availability

The original contributions presented in the study are included in the article, further inquiries can be directed to the corresponding author.
